# Human Herpesvirus‐6 Infectious Meningitis With Lymphadenitis in an Immunocompetent Adult

**DOI:** 10.1002/brb3.70590

**Published:** 2025-05-30

**Authors:** Yu Xie, Chenrui Zhang, Mingchi Ma, Ziqian Yin, Liqian Chen, Yimiao Yang, Shuang Wu, Yuanteng Fan, Yan Xu, Dan He

**Affiliations:** ^1^ Department of Neurology Zhongnan Hospital of Wuhan University Wuhan China

## Abstract

**Purpose:**

Human Herpesvirus 6 (HHV‐6) infections are primarily observed in immunocompromised individuals, such as those with acquired immunodeficiency syndrome or organ transplant recipients. However, its role as a pathogen in immunocompetent adults remains debated. We aimed to explore the clinical significance of HHV‐6 in immunocompetent individuals by presenting a case of HHV‐6‐associated meningitis with concurrent lymphadenitis.

**Method:**

We describe a case of an immunocompetent adult presenting with recurrent fever and headaches.

**Finding:**

Diagnostic evaluations included next‐generation sequencing analysis identified HHV‐6 in both cerebrospinal fluid specimens and lymphoid tissue samples. The patient demonstrated complete clinical resolution following a 14‐day course of ganciclovir therapy.

**Conclusion:**

This case underscores the need to consider HHV‐6 infection in immunocompetent adults presenting with meningitis of unknown etiology. Early detection and targeted antiviral therapy may lead to favorable clinical outcomes

## Graphical abstract

CSF: cerebrospinal fluid; NGS: next‐generation sequencing; HHV‐6: Human herpes virus‐6

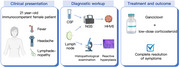

## 1 Introduction

Human herpes virus‐6 (HHV‐6), a member of the *β‐herpsvirinae* subfamily, is widely recognized for its ubiquity in childhood, affecting nearly all individuals at some point (Kim et al. 2020; Pandey et al. 2020). The HHV‐6 family consists of two distinct species, HHV‐6A and HHV‐6B, with the latter being more frequently associated with clinical manifestations in humans (Ablashi et al. 2014). Following primary infection, HHV‐6 establishes a lifelong latency within peripheral blood mononuclear cells, salivary glands, and neural tissues. Reactivation of the virus can occur in susceptible individuals, particularly those who are immunocompromised. In contrast, the pathogenicity of HHV‐6 in immunocompetent individuals remains controversial (Pandey et al. 2020; Kharbat et al. 2022; Pantry and Medveczky 2017). Cases of HHV‐6‐induced viral meningitis in immunocompetent adults are rare (Maniam et al. 2020; Alkozah et al. 2021; De Simone et al. 2013; Patel et al. 2021). The virus has also been implicated in cases of lymphadenitis, which can present diagnostic challenges due to its potential to mimic lymphoma (Balakrishna et al. 2018; Bai et al. 2014). Although prompt antiviral therapy is often effective, delays in treatment can result in poorer prognoses. The diagnosis of HHV‐6 infection remains challenging. However, with the advancement of diagnostic techniques, particularly the widespread use of next‐generation sequencing (NGS), the diagnostic rate for HHV‐6 infection has significantly improved.

This report describes a case of HHV‐6 meningitis complicated by lymphadenitis in a patient admitted to the Neurology Department of Zhongnan Hospital of Wuhan University in July 2024. The patient presented with recurrent episodes and nonspecific clinical symptoms. The diagnosis was confirmed through NGS analysis of cerebrospinal fluid (CSF) and lymph node tissue.

## 2 Clinical Data

A 21‐year‐old female presented to the Otolaryngology Clinic at Zhongnan Hospital of Wuhan University on June 18, 2024, with a 1‐week history of left‐sided cervical pain and low‐grade fever, unresponsive to a three‐day course of oral Umifenovir Hydrochloride and Oxacillin Sodium. Initial laboratory findings revealed leukopenia (white blood cell count: 2.80×10^9^/L; reference range 3.5–9.5×10^9^/L), neutropenia (absolute neutrophil count: 1.27×10^9^/L; 1.8–6.3×10^9^/L), and eosinopenia (Table 1). While chest radiography was unremarkable, ultrasonography demonstrated multiple enlarged cervical lymph nodes. Then the patient was admitted to the Otolaryngology‐Head and Neck Surgery Department with a preliminary diagnosis of acute cervical lymphadenitis. The patient's medical history was unremarkable for conditions such as hypertension, diabetes, hepatitis, tuberculosis, or other infectious diseases. Her immunization status was current according to the national vaccination schedule. Further laboratory examinations revealed elevated high‐sensitivity C‐reactive protein (11.14 mg/L; 0–3.0 mg/L), positive influenza A virus IgM antibody, and presence of Epstein‐Barr virus (EBV) DNA and EBV capsid antigen IgG. Following a three‐day course of oseltamivir, the patient's symptoms improved, and she was discharged.

**TABLE 1:** Complete blood count of the patient.

	White blood cell count (×10^9^/L)	Neutrophil count (×10^9^/L)	Lymphocyte count (×10^9^/L)	Monocyte counts (×10^9^/L)	Eosinophil count (×10^9^/L)
2024.06.18	2.80	1.27	1.24	0.28	0
2024.07.06	5.89	3.98	1.46	0.42	0
2024.07.27	4.00	3.10	0.50	0.40	0

**FIGURE 1:**
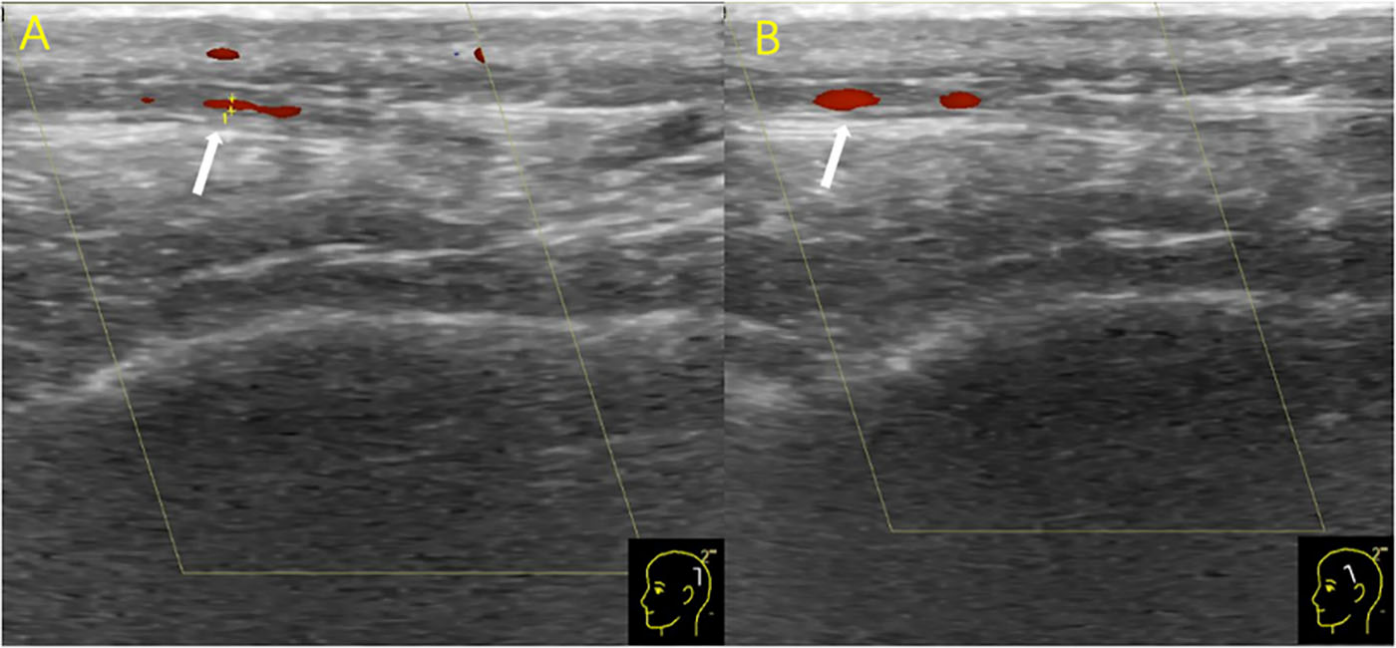
Ultrasound examination revealed the parietal branch (A) and frontal branch (B) of the left superficial temporal artery.

On July 6, the patient came to hospital with a 5‐day history of intermittent headaches and low‐grade fever. The headaches were characterized by intense, pulsating pain across the entire head, which exacerbated when squatting or lying down, and were accompanied by visual blurring, nausea, and vomiting. Laboratory blood tests revealed normal white blood cell, neutrophil, lymphocytes, and monocyte counts, with notable eosinopenia. Influenza virus antigen test was negative. CSF analysis demonstrated elevated protein level (0.68 g/L; 0.1–0.43 g/L) and pleocytosis (165 cells/µL; 0–8 cells/µL), comprising 97% mononuclear cells and 3% multinucleated cells (Table 2). NGS of the CSF detected Human Herpesvirus 6 type B (HHV‐6B) (30 reads, relative abundance of 0.25%). Ultrasonographic examination revealed intimal thickening in the parietal and frontal branch of the left superficial temporal artery (Figure 1). Based on the diagnosis of viral meningitis with arteritis, the patient was treated with oral Valacyclovir Hydrochloride and discharged with headache resolution.

**TABLE 2:** Cerebrospinal fluid analysis of the patient.

	Opening pressure (mmHg)	White blood cell count (cells/µL)	Protein (g/L)	Glucose (mmol/L)	Chloride (mmol/L)
2024.07.08	230	165	0.68	3.15	123.1
2024.07.28	200	30	0.33	3.27	124.5

**FIGURE 2:**
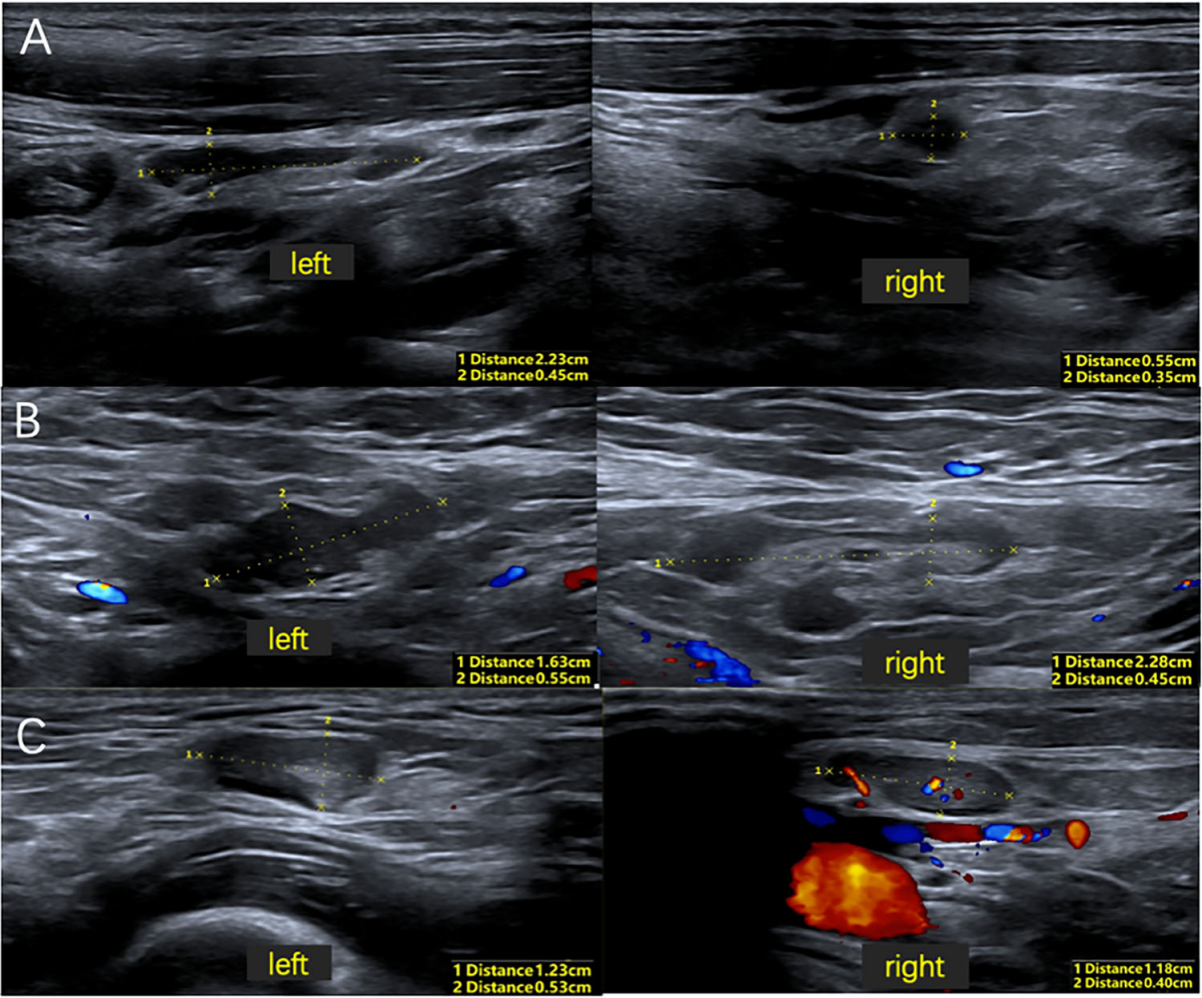
Ultrasound examination revealed enlargement of lymph nodes of bilateral cervical regions (A), axillary regions (B), and inguinal regions (C).

**FIGURE 3:**
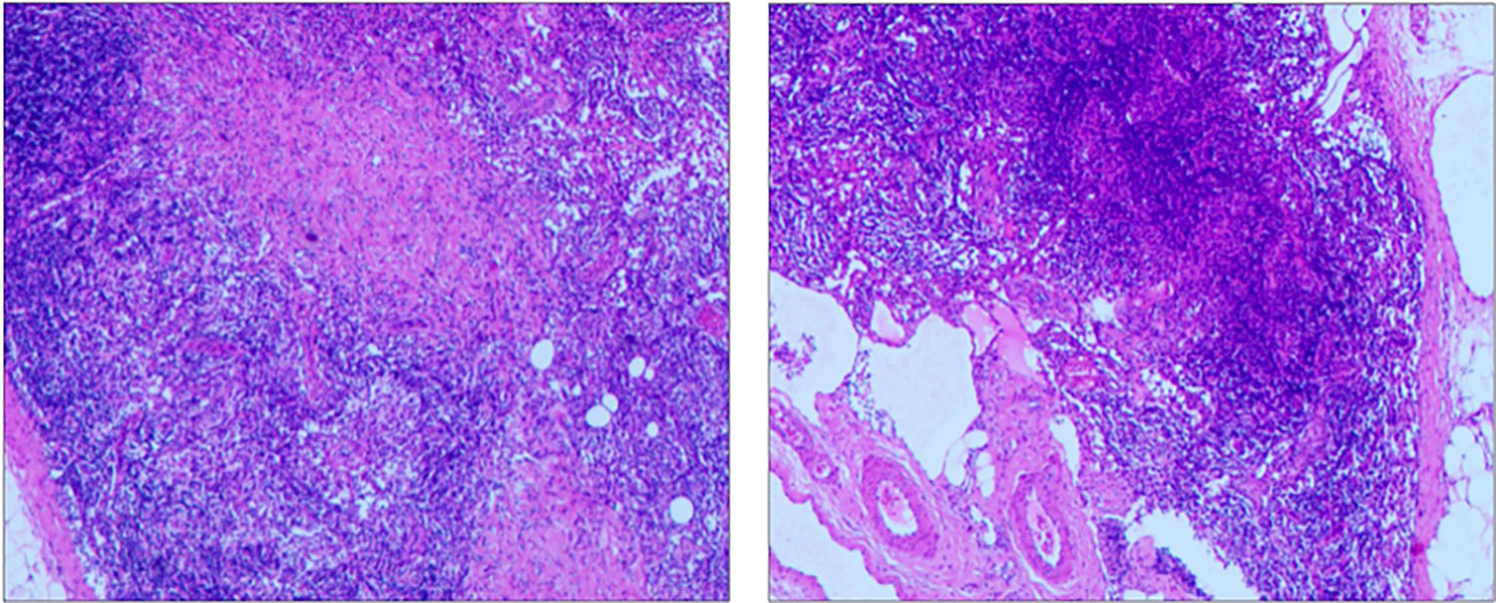
Histopathological examination of lymphoid tissue. The analysis indicated lymphoid reactive hyperplasia.

Twenty days post‐discharge, the patient was readmitted to the neurology department with recurrent fever, headaches, and general malaise. Neurological examination was unremarkable. Laboratory findings revealed lymphopenia and persistent eosinopenia. Serological testing was positive for both influenza virus A and B IgM antibodies. Blood cultures and EBV antibody test were negative. The lumbar puncture indicated a pressure of 200 mmH_2_O, normal CSF protein level (0.33 g/L), and elevated nucleated cell count (30 cells/µL) with predominantly mononuclear cells (93.3%). NGS of CSF yielded no identifiable pathogenic organisms. Autoimmune encephalitis antibodies were negative, and magnetic resonance imaging (MRI) findings were unremarkable. Ultrasound examination revealed enlargement of lymph nodes involving bilateral cervical, axillary, and inguinal regions (Figure 2). Histopathological examination of the right inguinal lymph node biopsy demonstrated reactive lymphoid hyperplasia (Figure 3). Immunohistochemical analysis was negative for HSVI, HSV II, CMV, and EBER. NGS of the lymph node tissue detected elevated levels of HHV‐6 type B (4 reads, relative abundance 0.68%) and human herpesvirus 7 (41 reads, relative abundance of 6.84%). Ultrasound imaging revealed no abnormalities in the abdominal aorta, renal artery, or subclavian artery. The patient received a 2‐week course of Ganciclovir Sodium combined with low‐dose corticosteroid therapy. Following treatment, the patient's headache resolved, and she was discharged afebrile. At 3‐month follow‐up, the patient remained asymptomatic.

## 3 Discussion

Based on the integrated analysis of clinical presentation, NGS results, and immunohistochemical findings, we diagnosed this case as HHV‐6‐associated meningitis with concurrent lymphadenitis. This case contributes to the limited literature documenting HHV‐6‐induced meningitis and lymphadenitis in immunocompetent adults (Maniam et al. 2020; Alkozah et al. 2021; De Simone et al. 2013; Patel et al. 2021; Balakrishna et al. 2018; Bai et al. 2014).

HHV‐6 was first isolated in 1986 from patients with lymphoproliferative disorders or HIV infection. Epidemiological studies indicate that by age of 2, HHV‐6 seroprevalence exceeds 80% in children (Okuno et al. 1989). Although latent infection or reactivation predominantly occurs in immunocompromised individuals (Agut et al. 2015), HHV‐6 has been associated with various clinical manifestations, including lymphadenitis, meningitis/encephalitis, encephalomyelitis, hepatitis, myocarditis, and pneumonia (Bai et al. 2014; Chang et al. 2009; Chia et al. 2024; Baleguli et al. 2021).

In the present case, the patient presented with recurrent fever and headaches, accompanied by ultrasonographic evidence of enlarged superficial lymph nodes, initially raised suggesting a potential neoplastic process, particularly lymphoma. However, subsequent lymph node biopsy and NGS analysis established the diagnosis of HHV‐related lymphadenitis.

Regarding the neurological symptoms, infectious or autoimmune meningitis was considered. The diagnosis of HHV‐6‐related meningitis was established based on NGS results and the absence of autoimmune encephalitis antibodies. Neurological manifestations of HHV‐6 infection include headache, drowsiness, coma, seizures, psychiatric disturbances, and focal neurological deficits (Kim et al. 2020). Traditional diagnostic methods for viral meningitis encompass viral culture, antigen detection, serological assays, and molecular biology techniques. Among these, molecular biology testing offering superior sensitivity and specificity facilitates early detection. In our patient, NGS proved crucial in identifying HHV‐6 in the CSF.

Given the scarcity of HHV‐6 meningitis in immunocompetent adults, there are no definitive treatment guidelines available. In vitro studies have demonstrated activity of ganciclovir, cidofovir, and foscarnet against HHV‐6 (Agut et al. 2017), although these findings may not necessarily predict clinical efficacy. Current guidelines recommend foscarnet or ganciclovir for HHV‐6 encephalitis treatment (Tunkel et al. 2008). Our patient initially received valacyclovir; however, symptom recurrence necessitated therapeutic modification. Following neurological department admission, a 2‐week ganciclovir course resulted in sustained symptom resolution.

In conclusion, HHV‐6 represents an uncommon etiology of meningitis and lymphadenitis in immunocompetent adults, often presenting with atypical features that may lead to diagnostic challenges. Timely therapeutic intervention is essential to prevent adverse outcomes and long‐term sequelae. The utilization of NGS technology substantially improves etiological identification in cases of meningitis and lymphadenitis, thereby improving patient management and outcomes.

## Author Contributions

**Yu Xie**: conceptualization, methodology, visualization, investigation, validation, formal analysis, writing – review and editing, writing – original draft. **Chenrui Zhang**: data curation, software, writing – original draft, writing – review and editing, visualization. **Mingchi Ma**: investigation, writing – original draft, writing – review and editing, methodology, software. **Ziqian Yin**: investigation, writing – review and editing, formal analysis, resources, data curation. **Liqian Chen**: writing – review and editing, methodology, investigation, visualization, formal analysis, data curation. **Yimiao Yang**: writing – review and editing, visualization, formal analysis, software, investigation, data curation. **Shuang Wu**: writing – review and editing, writing – original draft, validation, formal analysis, data curation, software, methodology. **Yuanteng Fan**: writing – review and editing, writing – original draft, investigation, methodology, visualization, formal analysis, data curation. **Yan Xu**: resources, supervision, project administration, validation, writing – review and editing, funding acquisition, conceptualization. **Dan He**: writing – review and editing, project administration, funding acquisition, validation, supervision, resources, conceptualization.

## Ethics Statement

Written informed consent has been provided by the patient for the case details and images to be published. Details of the case can be published without ethical committee approval.

## Informed Consent Statement

Informed consent was obtained from the subject involved in the study.

## Conflicts of Interest

The authors declare no conflict of interest.

### Peer Review

The peer review history for this article is available at https://publons.com/publon/10.1002/brb3.70590.

## Data Availability

The data that support the findings of this study are available from the corresponding author upon reasonable request.
